# Cholinergic Signaling, Neural Excitability, and Epilepsy

**DOI:** 10.3390/molecules26082258

**Published:** 2021-04-13

**Authors:** Yu Wang, Bei Tan, Yi Wang, Zhong Chen

**Affiliations:** 1 Key Laboratory of Neuropharmacology and Translational Medicine of Zhejiang Province, College of Pharmaceutical Science, Zhejiang Chinese Medical University, Hangzhou 310053, China; 18865516025@163.com (Y.W.); iris813704@163.com (B.T.); 2 Epilepsy Center, Department of Neurology, Second Affiliated Hospital, School of Medicine, Zhejiang University, Hangzhou 310058, China

**Keywords:** cholinergic, muscarinic, nicotinic, excitability, epilepsy

## Abstract

Epilepsy is a common brain disorder characterized by recurrent epileptic seizures with neuronal hyperexcitability. Apart from the classical imbalance between excitatory glutamatergic transmission and inhibitory γ-aminobutyric acidergic transmission, cumulative evidence suggest that cholinergic signaling is crucially involved in the modulation of neural excitability and epilepsy. In this review, we briefly describe the distribution of cholinergic neurons, muscarinic, and nicotinic receptors in the central nervous system and their relationship with neural excitability. Then, we summarize the findings from experimental and clinical research on the role of cholinergic signaling in epilepsy. Furthermore, we provide some perspectives on future investigation to reveal the precise role of the cholinergic system in epilepsy.

## 1. Introduction

More than 70 million people have epilepsy worldwide, accounting for about 1% of the population, which makes it one of the most common neurological conditions [1]. Epilepsy is usually characterized by recurrent seizures resulting from the hypersynchronous discharge of large populations of neurons in the brain [2]. The exact mechanism of epilepsy is still not well understood. Classically, it caused by an imbalance of “excitation–inhibition” that usually is closely related to the excitatory glutamatergic transmission and inhibitory γ-aminobutyric acidergic (GABAergic) transmission. Based on this classical theory, most current anti-epileptic drugs (AEDs) are mainly used to control seizures via blocking the activity of excitatory glutamatergic transmission or enhancing the activity of inhibitory GABAergic transmission. However, there are still 30% of patients who become drug-resistant to epilepsy [3]. Thus, the other alternative mechanisms underlying epilepsy and some new therapies are still being intensively sought.

The cholinergic system in the brain modulates neuronal excitability, synaptic transmission, and synaptic plasticity, playing a significant role in many physiological functions [4]. The evidence of cholinergic mechanisms involved in epilepsy emerged at the beginning of the 19th century [5]. Numerous studies have shown that the systemic administration of cholinergic agonists carbachol or pilocarpine have long been known to induce seizure activity [6]. Then, findings from both experimental and clinical research indicated dysfunctional cholinergic signaling in epilepsy. However, the causal role of cholinergic neurons in the generation, propagation, and control of seizures has limited data and many inconsistent reports recently. Notably, we recently found that the selective activation of cholinergic neurons in the medial septum (MS) by using optogenetics could produce obvious anti-seizure effects [7]. These data collectively support the notion that cholinergic signaling may play a critical but heterogeneous role in epilepsy. As the different functions of acetylcholine (ACh) hinge on the site of release, receptor subtype, and target neuronal population or neural circuit, gaining an in-depth understanding of cholinergic system potential mechanisms in epilepsy are necessary stepping stones to uncover their wide-ranging applications in the clinical arena for the treatment of epilepsy. In this review, we revisit the considerable recent progress in cholinergic modulation of epilepsy and propose an integrative perspective of its contribution to epilepsy.

## 2. Cholinergic Signaling for Modulating Neural Excitability

### 2.1. Cholinergic Signaling in the Brain

In the central nervous system (CNS), ACh acts as a neuromodulator released from key groups of cholinergic neurons, which mainly consist of two primary cell types including long-projecting cholinergic neurons and local cholinergic interneurons. The majority of cholinergic neurons in the mammalian brain are found in some parts of the forebrain and brainstem, the cholinergic interneurons within the striatum (caudate-putamen and nucleus accumbens), and the long-projecting cholinergic neurons within the basal forebrain (BF) (including the MS, the vertical (DBv) and horizontal limbs of the diagonal band of Broca (DBh), the nucleus basalis (NB) of Meynert) [8,9], medial habenula, laterodorsal tegmental nucleus (LDT), pedunculopontine tegmental nucleus (PPN) [10,11], and so on. In addition to these regions, smaller cholinergic neurons are also found in the cerebral cortex [12]. Cholinergic neurons in the MS and DB project mainly to the hippocampus, thalamic nuclei, and cortex; cholinergic neurons in the NB of Meynert project mainly to amygdala and cortex; cholinergic neurons in the habenula project mainly to brain stem nuclei; while cholinergic neurons in the LDT and PPN project mainly to thalamic nuclei, ventral tegmental area, and substantia nigra. These cholinergic neurons play important roles in many different types of brain function in neural circuit-specific manners.

**Figure 1:**
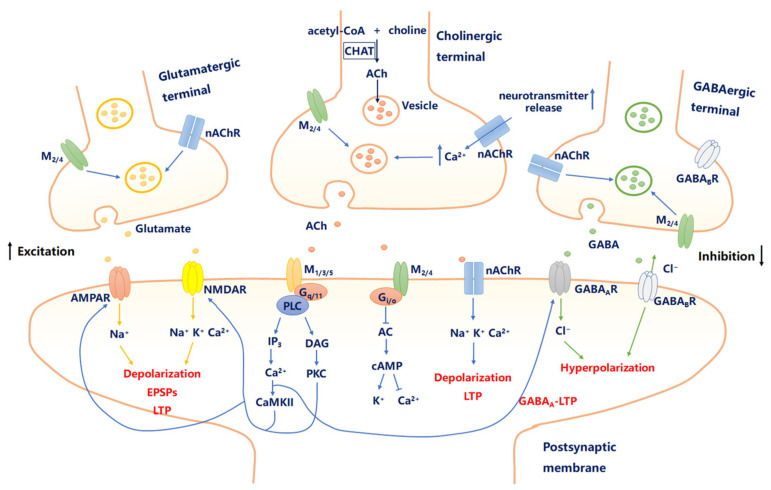
Cholinergic signaling modulates “excitation–inhibition” balance in the brain. Presynaptic or postsynaptic muscarinic acetylcholine receptors (mAChRs) and nicotinic acetylcholine receptors (nAChRs) influence synaptic plasticity by increasing intracellular Ca^2+^ release, induction of long-term potentiation (LTP), and leading to a depolarization. Additionally, the excitation of presynaptic nAChRs increases the release of many neurotransmitters including dopamine, norepinephrine, γ-aminobutyric acid (GABA), and glutamate (Glu) in a Ca^2+^-dependent manner.

ACh is synthesized in the cytosol of cholinergic terminals from choline delivered by a choline transport mechanism and acetyl-coenzyme A derived from the mitochondrial metabolism of pyruvate, which is acetylated by the enzyme choline acetyltransferase (ChAT). ChAT is responsible for ACh synthesis through the use of choline taken up by the high-affinity Na^+^-dependent choline transporter, and this is the rate-limiting factor in the synthesis process. Then, ACh is stored in synaptic vesicles with ATP before exocytotic release [13]. The storage of ACh by synaptic vesicles is mediated by the vesicular ACh transporter (VAChT), which catalyzes by the electrochemical gradient eliciting the exchanging of two vesicular protons for one cytoplasmic ACh [14]. Then, it is released from the nerve terminal by a Ca^2+^-dependent exocytotic process that occurs usually at the active zones in the presynaptic membrane. ACh released from cholinergic nerve terminals, often called ACh diffuse transmission, can spread to distant areas, and the concentration usually is less than 1 mM [15], but the multiple presynaptic impulses produce a sufficient release to activate the downstream signaling pathway via a variety of ACh receptors (AChRs). Central cholinergic receptors contain two superfamily groups: the G-protein-coupled muscarinic acetylcholine receptors (mAChRs, comprising five subtypes) family and the ligand-gated nicotinic ACh (nACh, comprising α and β subunits) channel family [16]. Finally, ACh is removed by the enzyme acetylcholinesterase (AChE) destruction (Figure 1).

### 2.2. mAChRs and Neural Excitability

There are five mAChRs encoding genes (M_1_-M_5_) that are generally divided into two broader groups based on their intracellular signaling cascades: postsynaptic M_1_, M_3_, and M_5_ receptors (M_1_-type receptor) are all coupled to G_q/11_ proteins that active phospholipase C (PLC) to generate inositol-1,4,5-triphosphate (IP_3_) and diacylglycerol (DAG), which resulted in intracellular stores to release Ca^2+^ and the activation of protein kinase C (PKC), respectively. Activated PKC subsequently phosphorylates GluA1 α-amino-3-hydroxy-5-methyl-4-isoxazole propionic acid receptors (AMPARs), which drives GluA1 into the synapse. Similarly, the activation of PLC facilitates IP_3_-induced Ca^2+^ release from intracellular Ca^2+^ stores, and the increase of Ca^2+^ activates Ca^2+^/calmodulin dependent protein kinase II (CaMKII), which is also considerable for reducing the excitability threshold, increasing the magnitude of LTP and the delivery of GluA1. In addition, M_1_ type receptor-mediated signaling affects neuronal excitability by closing voltage-gated K^+^ channels, blocking T-type Ca^2+^ channels, increasing the functional activity of glutamatergic receptors, and thus influx of cation such as Na^+^, K^+^, and Ca^2+^. For the other, M_2_ and M_4_ receptors (M_2_ type receptor) coupled to G_i/o_ inhibit the activity of adenylyl cyclase (AC) and thus reduce the availability of the second-messenger cyclic AMP. This links ACh activity to a variety of biochemical signaling cascades (Figure 1). The M_2_ type receptor also modulates ion channels: including suppressing the L-type voltage-gated calcium channel (VGCC) and opening the inward rectifying potassium channel (GIRK). M_2_ type receptor has been identified heterosynaptically as pre- and post-synaptic receptors, which alter both the intrinsic excitability of neurons and the release probability of various transmitters. Consequently, the M_1_ type receptor and M_2_ type receptor affect different downstream signaling and different second-messenger cascades, and they produce different effects on neural excitability.

The mAChRs critically contribute to the development of synaptic plasticity. mAChRs, expressing on both glutamatergic and GABAergic terminals to regulate the release of Glu and GABA, modulating hippocampal synaptic plasticity through intracellular signaling pathways downstream of mAChRs, which altered the subsequent response to excitatory inputs on both GABAergic and glutamatergic neurons in the hippocampus. The long-term potentiation (LTP) is expressed postsynaptically by a raise of AMPARs and *N*-methyl-d-aspartate glutamate receptors (NMDARs) or GABA_A_R response, and presynaptically via an increased Glu or GABA release probability, which are all induced by mAChRs signaling transduction. As we all know that postsynaptic mAChRs produce IP_3_, several neurotransmitters are triggering IP_3_ and inducing Ca^2+^ release from IP_3_-sensitive stores via VGCC, mediating the process of GABA_A_-LTP, which is a long-lasting potentiation of GABA_A_ inhibition [17] or long-term facilitation of AMPAR-mediated transmission. ACh, M_1_ receptors, and IP_3_R share a parallel LTP of AMPARs- and NMDARs-mediated transmission [18]. Furthermore, the activation of M_1_ receptors serves as a metaplastic switch making inhibitory synapses LTP induced by glutamatergic synapses [19]. Moreover, mAChRs contribute to the neuronal rhythm in the brain. The mAChRs agonist carbachol depolarizes in hippocampal interneurons, inducing θ-frequency membrane potential oscillations [20]. Indeed, the optogenetic stimulation of septal cholinergic neurons modulates interneuron excitability and increases spontaneous activity [21]. In summary, mAChRs can influence the intrinsic excitability of neuron and synaptic transmission as well as synaptic plasticity.

### 2.3. nAChRs and Neural Excitability

As mentioned above, neuronal nAChRs is a pentameric-composed ion channel that is permeable to cations, which lead Na^+^, K^+^, and Ca^2+^ ions across the membrane, while the subunits that are assembled into neuronal nAChRs include α2-α10 and β2-β4, encoded respectively by the *CHRNA2*-*CHRNA10* and *CHRNB2*-*CHRNB4* genes [4]. nAChRs subtypes localize to presynaptic and postsynaptic, which regulates both presynaptic and postsynaptic processes involved in neuronal excitability and synaptic plasticity by fostering neurotransmitter release or downstream signal transduction. nAChRs binded by ACh, lead to membrane depolarization followed by the excitation of presynaptic or postsynaptic terminals while stimulating neurotransmitter release or action potential transmit (Figure 1). Stimulation of presynaptic nAChRs can increase the release of Glu, GABA, ACh, 5-HT, et al., which is dependent on brain regions. Stimulation of postsynaptic nAChRs can induce significant inward currents in neurons in many brain regions, including the hippocampus and cortex.

The nAChRs also significantly contribute to the development of synaptic plasticity. Nicotine could negatively modulate the function of AMPARs present on glutamatergic nerve terminals in the rat trigeminal caudal nucleus. Dynamic control of AMPARs by nAChRs can result in LTD and LTP [22]. In the CNS, α4β2 and α7 nAChRs are the two predominant nAChRs subtypes in the brain. The stimulation of presynaptic α4β2 or α7 nAChRs depolarizes hippocampal interneurons, indirectly affects neurotransmitters such as Glu or GABA release by activating VGCC, boosts the release of Ca^2+^, and enhances the induction of LTP or frequency of miniature excitatory postsynaptic currents [23]. Consistent with postsynaptic mAChRs findings, α7 nAChRs is highly permeable to Ca^2+^, and its activation increases intracellular Ca^2+^ and leads to CaMKII activation, which also contributes to the ACh-triggered synaptic GluA1 delivery [24]. Similarly, presynaptic or postsynaptic nAChRs influence synaptic plasticity by leading a depolarization and increasing an intracellular Ca^2+^ release, which relieves the Mg^2+^ block of postsynaptic NMDA receptors and promotes to transmit glutamatergic or GABAergic transmission, which then enhances excitatory transmission to higher centers [25]. Recent evidence indicates that heterologous α4β2 nAChRs have been shown to be localized to the presynaptic membrane [26]. The nicotine increased the activity of glutamatergic neurotransmission, and the concentration of Glu through the activation of presynaptic α4β2 nAChRs induced dendritic spine enlargement [27]. The activation of presynaptic α3β4 nAChRs in parvalbumin-positive cells stimulates tetrodotoxin-insensitive GABA release via T-type VGCC and Ca^2+^ from internal stores [28]. A study proved that α7 nAChRs act as a bidirectional role in modulating network excitability in the prelimbic cortex. Both global activation (by α7 nAChRs positive allosteric modulator PNU-120596 and agonist PNU-282987) and inhibition (by the α7 nAChRs antagonist methyllycaconitine (MLA) of α7 nAChRs inhibited the induction of θ-burst-induced LTP, indicating the diverse effects of α7 nAChRs on excitation and inhibition [29]. Hence, the prior studies collectively demonstrated that neuronal nAChRs regulate both excitatory and inhibitory transmission as mAChRs, which is often subtype-specific, varying across different brain areas. This is fundamental to understanding the role of cholinergic receptors in neural excitability and plasticity.

## 3. Cholinergic Signaling in Epilepsy

Given the outstanding roles of ACh in the modulation of neural excitability by binding different subtypes of AChRs, it is not surprisingly that cholinergic dysfunction is closely correlated with epilepsy, which is mainly caused by the imbalance of excitation and inhibition. Then, we will mainly focus our discussion on the relationship between cholinergic signaling and epilepsy.

### 3.1. mAChRs and Epilepsy

A study suggested that there was a reduction of ChAT activity and mAChRs binding in the piriform cortex, amygdala, and nucleus basalis, a decrease of AChE activity in the piriform cortex, and a loss of Na^+^-dependent high-affinity choline uptake in the piriform cortex and amygdala in a kainic acid-induced epilepsy model [30]. Furthermore, clinical data demonstrated that the binding of mAChRs antagonist I-iododexetimide was decreased in the anterior hippocampus in partial seizures patients [31]. These reflect the dysfunction of cholinergic signaling in epilepsy.

Muscarinic excitation contributes to increasing susceptibility to epileptogenesis and rewiring hippocampal circuitry [32]. Importantly, pilocarpine, which is an mAChRs agonist, is one of the most common used chemicals to induce seizure models. Of the mAChRs, M_1_ is among the most heavily expressed in the forebrain and midbrain regions and has been proposed to play key roles in the regulation of epilepsy. A study using mice with deletion of the five muscarinic receptor subtypes made several observations; only M_1_ KO mice did not display seizures and survived after pilocarpine administration, while M_2_-M_5_ KO mice all displayed clonic seizures and died within 60 min after pilocarpine administration [33]. It is noteworthy that the mAChRs agonist pilocarpine does not evoke seizure activity in both homozygous and heterozygous M_1_ mutant mice [34], which suggests that the role of the M_1_ subtype mediates pilocarpine-induced seizures. Moreover, M_1_ mAChRs are vital to γ generation, which is thought to be generated by PV cells and precedes seizure onset [35]. Molecular pharmacology studies indicate that the M_1_ mAChRs selectivity antagonist VU0255035 is efficacious in reducing pilocarpine-induced seizures in mice [36] and holding over the process of status epilepticus after organophosphates (OPs) such as paraoxon or soman exposure [37]. Together, those data suggested that M_1_ mAChRs may be the important subtype in the regulation of epileptic seizures.

M_2_ receptors always increased in various epilepsy models including febrile seizure, hippocampal sclerosis, and other neocortical pathologies [38]. Recent studies have shown that the increase of M_2_ mAChRs in the brainstem in pentylenetetrazole (PTZ)-kindled epileptic rats [39]. In vitro receptor binding studies have further shown an enhancement of M_2_ receptors binding in the lateral amygdala nuclei of TLE patients, while binding to M_3_ receptors was reduced [40]. Intriguingly, there is no obvious seizure phenotype in global M_2_ knockout mice [33]. Additionally, sparteine, an anticonvulsant drug, increasing the hippocampal M_4_ receptor expression on PTZ-induced seizures, indicating that the M_4_ receptor may be also critical for seizures [41].

As previously indicated, limbic and brainstem systems are two important anatomical systems involved in epileptic seizures, leading to the limbic seizure and the brainstem seizure. Intrahippocampal and intracerebroventricular administration of mAChRs agonists in rats produced sustained limbic seizures and brain damage. Intrahippocampal pilocarpine or bethanechol administration-induced limbic seizures are initiated via mAChRs and further mediated excitotoxicity via NMDARs [42]; EEG in CA3 showed spiking activity of high frequency, with rapid propagation to the lateral septum, amygdala, and neocortex along with the hippocampus [43]. Intraamygdaloid administration of kainic acid elicits epileptiform electroencephalographic activity; subsequently, neuronal loss and gliosis were marked at the various hippocampal fields, the midline thalamic nuclei, lateral septum, and cortical areas [44]. Furthermore, ACh may tonically enhance the excitability of cerebral cortical neurons, which might account for an increase in the effectiveness of other excitatory inputs and facilitate the development of epileptogenesis [45]. These results suggest that the overstimulation of mAChRs leads to limbic seizures.

Tonic-clonic seizure is considered to be mediated by brainstem structures. A microinjection of carbachol into the nucleus reticularis pontis oralis, one of the brainstem structures, inhibited the maximal electroshock seizure (MES) in rats [46]. A microinjection of carbachol into the periaqueductal gray (PAG) region of rats induced seizure behavior accompanied by epileptiform electrocorticogram afterdischarge recorded from the parietal cortex. Interestingly, limbic seizure activity, similar to amygdala-kindled seizures was also induced in two animals. In addition, a PAG microinjection of bicuculline induced clonic seizures, myoclonic activity, or limbic seizures [47]. These reports indicate that the mAChRs signaling in the brainstem system may regulate the limbic seizure and brainstem seizure activity collectively.

Despite there already being various studies about the role of mAChRs in epileptic seizures (Table 1), how mAChRs are involved in the different stages of epilepsy still needs to be investigated.

### 3.2. nAChRs and Epilepsy

The systemic or central administration of α7 antagonist MLA is known to block nicotine-induced seizures in mice [48,49], and choline dose-dependently ameliorated seizure severity in PTZ-kindled mice [50]. A previous study tested the anti-seizure activity of various novel amino-alkyl-cyclohexane derivatives, among which nAChRs antagonists have shown an overlap potency between channel blocking at nAChRs and NMDARs. nAChRs preferring antagonists strongly relieved MES and nicotine-induced seizure in mice. The effect of anticonvulsant in the MES was reduced by an additional injection of a subconvulsant dose of nicotine. However, such efficacious anticonvulsants were not observed in kindled rats [51]. These indicated that nAChRs antagonists might be a promising therapeutic approach to treat generalized seizures rather than complex partial seizures. Furthermore, α7 nAChRs currently were found to regulate the hyperfunction of glutamatergic synaptic transmission in the hippocampus samples obtained from patients with mesial temporal lobe epilepsy with hippocampal sclerosis [52].

As described for a variety of nocturnal epilepsy syndromes, autosomal dominant sleep-related hyper motor epilepsy (ADSHE) predominantly related to sleep, and approximately 12% of the ADSHE families carry mutations on genes coding for subunits of the neuronal nAChRs (major subtypes: homomeric α7 and heteromeric α4β2). To date, ADSHE mutations are mainly in *CHRNA2* (α2^I279N^), *CHRNA4* (α4^S248F^, α4^S252L^, α4^T265I^, α4^776ins3^), and *CHRNB2* (β2^V287M^, β2^V287L^, β2^I312M^, β2^L301V^, β2^V308A^). A previous study suggested that the modulation of α4β2 nicotinic receptors plays a role not only in ADSHE but also in other genetic epileptic syndromes such as idiopathic generalized epilepsy and could serve as a biomarker of epilepsy syndromes with a genetic background. The mutant in β2^V287L^ presynaptic nAChRs triggering neuronal firing, serving as an enhancement of neurotransmitter release or the abnormal mutant in postsynaptic nAChRs that may cause hyperexcitability [53]; β2^V287L^ also causes spontaneous seizures during periods of increased δ wave activity [54]. Interestingly, the previous study suggested that the treatment of carbamazepine (CBZ) in ADSHE is mainly through nAChRs, which is supported by the evidence that 100 μM CBZ inhibits ACh-evoked currents at the human α4β2 nicotinic receptors and the ADSHE α4^S248F^ or α4^L-776ins3^ mutant receptors with 3-fold increase in sensitivity to CBZ [55]. Additionally, an increase of midbrain nAChRs density could be involved in the pathological of ADSHE through the brainstem cholinergic signaling in the ascending arousal system [56]. A study of ADSHE variants in *CHRNB2* and *CHRNA4* closely relevant to patients with insular epilepsy recently, *CHRNB2* and *CHRNA4* increased nicotinic currents in whole-cell recording [57]. In addition, clinical data demonstrated that mutations in *CHRNA4* may be a novel gene causing genetic or focal epilepsy with febrile seizures [58] and familial partial epilepsy with variable foci [59], it aims to broaden the genotypic-phenotypic spectrum of combined epileptic in *CHRNA4*.

In addition, an experimental study has demonstrated that cholinergic systems closely linking to the pathogenesis of Rett syndrome (RTT), and RTT patients suffer from epilepsy up to 80% [60]. Mutations in the X-linked gene encoding the transcriptional regulator *Mecp2* cause RTT. Conditional deletion of *Mecp2* in cholinergic neurons resulted in the alteration of epilepsy susceptibility, which could be relieved by re-expressing *Mecp2* in the BF cholinergic neurons of *Chat-Mecp2^−/y^* mice, which implicated the relationship of BF cholinergic system and epilepsy. *Chat-Mecp2^−/y^* mice displayed frequent hyperexcitability discharges. Furthermore, the administration of α7 nAChRs agonist PNU282987 in the CA1 of the hippocampus increased the seizure onset time [61]. These findings collectively proved that the dysfunction of cholinergic neurons can contribute to epilepsy through nAChRs (Table 2).

Similar as mAChRs, nAChRs are also implicated in the pathogenesis of the different type of epilepsy. Nicotine induced seizures by activating hippocampus and amygdalar neurons mainly via α7 nAChRs [49]. In humans, nAChR mutations associated with ADSHE seizures occur in the frontal lobe [54]. These results suggest that the overstimulation of nAChRs leads to limbic seizures, while intra-inferior colliculus microinjection of different doses in nAChRs antagonists has a different role in the modulation of spontaneous seizures [62]. It indicated the nAChRs may also contribute to brainstem seizures. The role of the cholinergic system in epilepsy has long been studied, but most researchers have focused on mAChRs rather than nAChRs, and the role of nAChRs is yet to be further invested in limbic seizure and the brainstem seizure.

### 3.3. Cholinergic Neurons Circuit in Epilepsy

**Figure 2:**
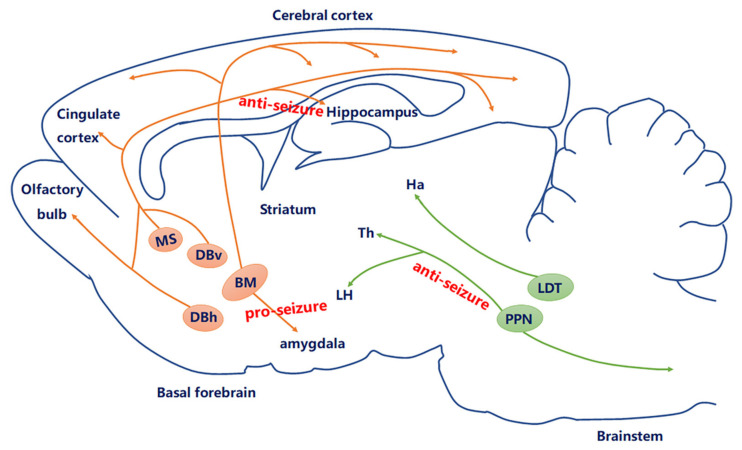
Current knowledge of the role of the cholinergic circuit in epilepsy. The central cholinergic afferents mostly originate from the basal forebrain (BF), including medial septum (MS), DBv, DBh, and nucleus basalis (NB). Another derives from the brain stem, including the laterodorsal tegmental nucleus (LDT) and pedunculopontine tegmental nucleus (PPN). Currently, studies have proven that MS–hippocampal cholinergic neurons produced anti-seizure effects; optogenetic stimulation of PPN cholinergic neurons may be a new way to regulate cortical dysfunction during epileptic seizures through subcortical arousal networks; amygdala cholinergic connections to the BF may contribute to epilepsy.

Currently, studies of clinical and experimental models of epilepsy suggest a more precise conceptual mechanism that seems to underlie epilepsy: a change in the excitation–inhibition (E-I) balance in circuit-level dysfunction. Therefore, exploring and understanding the precise circuit-level cholinergic mechanism of epileptic seizures is essential for precise circuit therapy and regulation. The question arises as to how cholinergic neurons modulate the circuit-level dysfunction of an epileptic seizure. Epilepsy originates from the limbic system, especially from amygdaloid and hippocampal regions. Numerous studies have attempted to explain how the BF cholinergic system projects to the cortex [63], hippocampus, and amygdala [64,65], which are critical regions for the seizure generation and spread (Figure 2). For example, the changes in the amygdaloidal cholinergic connections from the BF may contribute to epilepsy-related hyperexcitability [50]. Nicotine elicits convulsive seizures by activating amygdala neurons mainly via α7 nACh receptors. Intracerebrally, physostigmine, a reversible cholinergic medication, in limbic structures has been reported to prolong seizure by increased sensitivity to kindling stimulations [66]. However, intraventricular administration of 192 Immunoglobulin G -saporin, which inhibits cholinergic projection to the hippocampus and cortex respectively, facilitates seizure induced by amygdala kindling [67]. These data suggested that cholinergic neurons may play a critical but heterogeneous role in epilepsy at the circuit level.

The hippocampus that receives cholinergic projection from the BF has been a particular focus in the study of TLE. In TLE, ACh regulates the spread of excitatory activity within hippocampal and cortical circuits during the seizure; seizures are initiated in the hippocampus or entorhinal cortex (EC) due to the dysfunction of cholinergic tone. The cholinergic tone modulates ongoing hippocampal activities by enhancing excitatory and depressing inhibitory transmissions, thus increasing excitatory output to the EC to further promote θ generation in the EC–hippocampal network [68]. Recently, the selective activation of MS–hippocampal cholinergic neurons enhanced θ rhythm and suppressed peri-θ frequency bands, creating sharp-wave ripples [21]. We found that MS cholinergic neurons ceased firing during hippocampal seizures. Optogenetic stimulation of the MS–hippocampal cholinergic circuit reduces hippocampal seizures. This anti-seizure effect was mediated by the hippocampal somatostatin neuron, as the chemogenetic inhibition of hippocampal somatostatin-positive (rather than parvalbumin-positive) subtype of GABAergic neurons reversed the antiseizure effect of the MS–hippocampus cholinergic circuit [7]. Collectively, these data suggested that the BF–hippocampal cholinergic circuit has been implicated in the pathophysiology of epilepsy and may be a promising anti-seizure target. However, it is still unclear whether the activation of cholinergic projections to other ictogenic regions of the temporal lobe suppresses seizures. Importantly, we previous showed that the hippocampal subiculum is an important gate for seizure generalization and drug-resistant states [69,70,71,72], but whether cholinergic input within the subiculum is involved in seizure modulation need further study. It is possible that regional differences in the role of ACh could begin to explain the discrepancy between the anticonvulsant effects in our study and the proconvulsant effects reported in other models.

Additionally, the brainstem PPN contains cholinergic neurons and provides the bulk of the cholinergic input to the thalamus, particularly to its relay and reticular nuclei, which is centrally involved in attention or arousal [73]. Prior work demonstrates a decrease in the levels of choline of both the thalamus and cortex for reduced subcortical arousal during partial seizures. Moreover, the hyperpolarization of PPN and BF cholinergic neurons and reduction of excitatory synaptic input and firing are accompanied by a decrease in EPSP-like activity during focal limbic seizures [10,11]; limbic seizures also caused cortical low-frequency oscillations by inhibiting cholinergic arousal systems in the forebrain. All these data support the possible cellular mechanism of decreased subcortical cholinergic arousal in focal seizures by improving cognition. Interestingly, there is an enhancement in cortical γ activity and a depression in δ activity in response to the selective activation of cholinergic brainstem neurons in the PPN during focal hippocampal seizures [74], which implicated that optogenetic stimulation of subcortical arousal networks may be a new means to moderate cortical dysfunction during epileptic seizures.

The prefrontal cortex (PFC) is known to play an essential role in epileptic activity. Intracerebral microinjection of carbachol into the medial PFC of rats induced a high amplitude spiking representative of seizures, which is accompanied by an atypical form of seizures [75,76]. The PFC cholinergic projections can boost the γ rhythms local networks and regulate the early activity within PFC–hippocampal circuits [77]. As previously suggested, limbic thalamic nuclei amplified seizures from the temporal hippocampal formation to the PFC [78,79], whereas it remains unknown how the cholinergic projection of PFC–hippocampal circuits interact with seizure modulation.

The inferior colliculus is the initiation site for acoustically evoked seizures (or audiogenic seizures, AGS). Previously, studies demonstrated that an intracellular microinjection of intermediate doses of nAChRs antagonists decreased the threshold current of seizure initiation, while higher doses of the nAChRs antagonists caused spontaneous seizures [62]. Additionally, a study indicated that microinjections of carbachol into the IC elicited myoclonic seizures, and microinjections of the gallamine into the IC induced AGS susceptibility [80]. Nevertheless, the correlation between cholinergic circuit in the IC and epilepsy is still unclear.

Likewise, the giant cholinergic interneurons of the striatum regulate several aspects of basal ganglia function, which mainly affects motor function. Relative to other brain areas, the striatum contains higher levels of ACh, as well as both mAChRs and nAChRs that mediate its presynaptic and postsynaptic effects [81]. However, the relationship between cholinergic neurons of the striatum and seizure is still largely unknown.

## 4. Conclusions and Outlook

Here, we have presented a selected review of recent work on cholinergic dysfunction in epilepsy at molecular, cellular, and circuit levels. Some of the issues that have been discussed remain quite controversial, especially the difference from molecular and circuit levels. Currently, we are still at the infant stage in regard to the precise circuit mechanism of the cholinergic system in epileptic seizures. With the rapid development of modern technologies of optogenetics, trans-synaptic viral tracing, single-unit recordings, and two-photon microscopy for cell and circuit-specific modulation, understanding the cholinergic circuit mechanisms of epilepsy is becoming a reality and an easy job, and circuit-level therapy targeting cholinergic neuron is a promising and potentially more precise option for epileptic treatment in the future.

## Figures and Tables

**Table 1:** Summaries of findings reporting the role of mAChRs in epilepsy.

Epilepsy Model	Time Point	Observations	References
Temporal lobes with complex partial seizures	Interictal period	The binding of mAChRs antagonist I-iododexetimide was decreased in the anterior hippocampus.	[31]
Patients with drug-resistant focal temporal lobe epilepsy	Interictal period	M_2_ receptors always increased in various seizures including febrile seizure, hippocampal sclerosis, and other neocortical pathologies.	[38]
Patients with intractable temporal lobe epilepsy	Interictal period	An enhancement of M_2_ receptors binding in the lateral amygdala nuclei of TLE patients, while binding to M_3_ receptors was reduced.	[40]
Kainic acid	3 days after injection of kainic acid	1. The reduction of ChAT activity in the piriform cortex, amygdala, and nucleus basalis. 2. The reduction of AChE activity in the piriform cortex. 3. The decrease of mAChRs binding in the piriform cortex, amygdala, and nucleus basalis. 4. The decrease of Na^+^-dependent high-affinity choline uptake in the piriform cortex and amygdala.	[30]
Pilocarpine	30 min after administration of pilocarpine	1. M_1_ KO mice did not display seizures and survived after pilocarpine administration. 2. M_2_-M_5_ KO mice all had a seizure (clonic seizures) and died within 1 h after pilocarpine administration.	[33]
Pilocarpine	45 min after administration of pilocarpine	The inability of pilocarpine to evoke seizures in both homozygous and heterozygous M_1_ mutant mice.	[34]
Pilocarpine	45 min after administration of pilocarpine	1. VU0255035 suppresses the potentiation of NMDAR currents induced by carbachol in hippocampal pyramidal cells. 2. VU0255035 inhibits pilocarpine-induced seizures.	[36]
OPs	60 min after administration of OPs	VU0255035 retarded the process of status epilepticus after OPs exposure.	[37]
PTZ kindling model	30 min after administration of PTZ	The increase of M_2_ receptors was observed in PTZ-kindled in the brainstem.	[39]
PTZ kindling model	180 and 240 min after administration of PTZ	Sparteine increases the hippocampal M_4_ receptor expression.	[41]

**Table 2:** Summaries of findings reporting the role of nAChRs in epilepsy.

Epilepsy Model	Time Point	Observations	References
Patients with mesial temporal lobe epilepsy with hippocampal sclerosis	Interictal period	α7 nAChRs were found to regulate hyperfunction of glutamatergic synaptic transmission in the hippocampus.	[52]
HEK293 cells co-expressing the human α4 nAChRs and the wild-type and the V287L mutant patient	-	1. The mutant in β2^V287L^ presynaptic nAChRs triggering neuronal firing, serving as an enhancement of neurotransmitter release. 2. The abnormal mutant in postsynaptic nAChRs may cause hyperexcitability.	[53]
Reconstituted in *Xenopus oocytes*	-	100 μM CBZ inhibits ACh-evoked currents at the human α4β2 nicotinic receptors, and the ADSHE α4^S248F^ or α4^L-776ins3^ mutant receptors, with a roughly 3 fold increase in sensitivity to CBZ.	[55]
ADSHE patients	Interictal period	An increase of midbrain nAChRs density in the ADSHE.	[56]
Patients with insular epilepsy	Interictal period	Mutant nACh receptors increased nicotinic currents in whole-cell recording.	[57]
Genetic or focal epilepsy with febrile seizures (GEFS+) patients	Interictal period	*CHRNA4* was the pathogenic gene of GEFS+.	[58]
Familial partial epilepsy with variable foci (FPEVF) patients	Interictal period	cHRNA4 was the pathogenic gene of FPEVF.	[59]
Nicotine	Intraperitoneally injected 15 min before the nicotine treatment.	Nicotine elicits convulsive seizures by activating amygdalar neurons mainly via α7 nACh receptors.	[48]
PTZ kindling	Exposed to PTZ injections on day 3, 6, and 9 of treatment to assess seizure severity score.	The amelioration of epilepsy by α7 nAChRs agonist choline chloride in PTZ-kindled mice model.	[50]
MES and nicotine-induced seizure test in mice;Amygdala-kindling in rats.	1.Nicotine-induced seizure starting immediately after nicotine injection and up to 5 min afterwards. 2. MES and kindling assesed interictal period.	1. Various novel amino-alkyl-cyclohexane derivatives, among which nAChRs antagonists have shown an overlap potency between channel blocking at nAChRs and NMDARs. 2. nAChRs preferring antagonists were strongly relived MES and nicotine-induced seizure in mice. 3. The effect of anticonvulsant in the MES was all reduced by an additional injection of a subconvulsant dose of nicotine. 4. Such efficacious anticonvulsants were not affected in kindled rats	[51]
Pilocarpine	EEG activities recorded 7 days post-surgical recovery	1. *Chat-Mecp2^−/y^* mice displayed frequent hyperexcitability discharges. 2. Administration of pilocarpine produces status epilepticus in *Chat-Mecp2^−/y^* mice. 3. Administration of α7 nAChRs agonist PNU282987 in the CA1 of the hippocampus increased the seizures onset time.	[61]

## Data Availability

Our study did not report any data.
